# Maxwell's demon in biochemical signal transduction with feedback loop

**DOI:** 10.1038/ncomms8498

**Published:** 2015-06-23

**Authors:** Sosuke Ito, Takahiro Sagawa

**Affiliations:** 1 Department of Physics, The University of Tokyo, Tokyo 113-0033, Japan; 2 Department of Basic Science, The University of Tokyo, Tokyo 153-8902, Japan

## Abstract

Signal transduction in living cells is vital to maintain life itself, where information transfer in noisy environment plays a significant role. In a rather different context, the recent intensive research on ‘Maxwell's demon'—a feedback controller that utilizes information of individual molecules—have led to a unified theory of information and thermodynamics. Here we combine these two streams of research, and show that the second law of thermodynamics with information reveals the fundamental limit of the robustness of signal transduction against environmental fluctuations. Especially, we find that the degree of robustness is quantitatively characterized by an informational quantity called transfer entropy. Our information-thermodynamic approach is applicable to biological communication inside cells, in which there is no explicit channel coding in contrast to artificial communication. Our result could open up a novel biophysical approach to understand information processing in living systems on the basis of the fundamental information–thermodynamics link.

## Abstract (web summary)

The connection between information and thermodynamics is embodied in the figure of Maxwell's demon, a feedback controller. Here, the authors apply thermodynamics of information to signal transduction in chemotaxis of *E. coli*, predicting that its robustness is quantified by transfer entropy.

## Graphical abstract

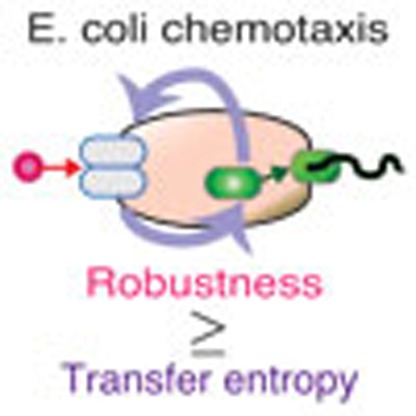

A crucial feature of biological signal transduction lies in the fact that it works in noisy environment^1^,^2^,^3^. To understand its mechanism, signal transduction has been modelled as noisy information processing^4^,^5^,^6^,^7^,^8^,^9^,^10^,^11^. For example, signal transduction of bacterial chemotaxis of *Escherichia coli* (*E. coli*) has been investigated as a simple model organism for sensory adaptation^12^,^13^,^14^,^15^,^16^. A crucial ingredient of *E. coli* chemotaxis is a feedback loop, which enhances the robustness of the signal transduction against environmental noise.

The information transmission inside the feedback loop can be quantified by the transfer entropy, which was originally introduced in the context of time series analysis^17^, and has been studied in electrophysiological systems^18^, chemical processes^19^ and artificial sensorimotors^20^. The transfer entropy is the conditional mutual information representing the directed information flow, and gives an upper bound of the redundancy of the channel coding in an artificial communication channel with a feedback loop^21^; this is a fundamental consequence of Shannon's second theorem^22^,^23^. However, as there is not any explicit channel coding inside living cells, the role of the transfer entropy in biological communication has not been fully understood.

The transfer entropy also plays a significant role in thermodynamics^24^. Historically, the connection between thermodynamics and information was first discussed in the thought experiment of ‘Maxwell's demon' in the nineteenth century^25^,^26^,^27^, where the demon is regarded as a feedback controller. In the recent progress on this problem in light of modern non-equilibrium statistical physics^28^,^29^, a universal and quantitative theory of thermodynamics feedback control has been developed, leading to the field of information thermodynamics^24^,^30^,^31^,^32^,^33^,^34^,^35^,^36^,^37^,^38^,^39^,^40^,^41^,^42^,^43^,^44^,^45^,^46^,^47^,^48^. Information thermodynamics reveals a generalization of the second law of thermodynamics, which implies that the entropy production of a target system is bounded by the transfer entropy from the target system to the outside world^24^.

In this article, we apply the generalized second law to establish the quantitative relationship between the transfer entropy and the robustness of adaptive signal transduction against noise. We show that the transfer entropy gives the fundamental upper bound of the robustness, elucidating an analogy between information thermodynamics and the Shannon's information theory^22^,^23^. We numerically studied the information-thermodynamics efficiency of the signal transduction of *E. coli* chemotaxis, and found that the signal transduction of *E. coli* chemotaxis is efficient as an information-thermodynamic device, even when it is highly dissipative as a conventional heat engine.

## Results

### Model

**Figure 1:**
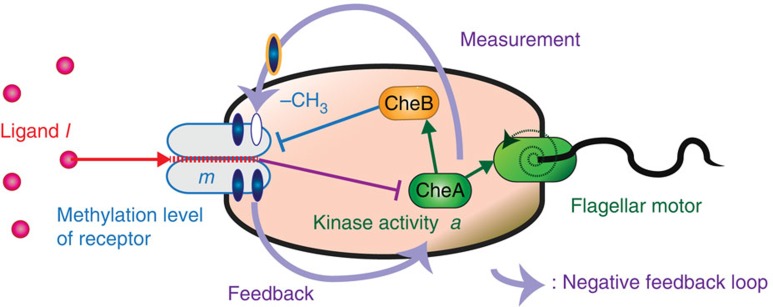
Schematic of adaptive signal transduction of *E. coli* bacterial chemotaxis. Kinase activity *a* (green) activates a flagellar motor to move *E. coli* towards a direction of the higher ligand density *l* (red) by using the information stored in methylation level *m* (blue). CheA is the histidine kinase related to the flagellar motor, and the response regulator CheB, activated by CheA, removes methyl groups from the receptor. The methylation level *m* plays a similar role to the memory of Maxwell's demon 8,24, which reduces the effect of the environmental noise on the target system *a*; the negative feedback loop (purple arrows) counteracts the influence of ligand binding.

The main components of *E. coli* chemotaxis are the ligand density change *l*, the kinase activity *a* and the methylation level *m* of the receptor (Fig. 1). A feedback loop exists between *a* and *m*, which reduces the environmental noise in the signal transduction pathway from *l* to *a* (ref. 49). Let *l*_*t*_, *a*_*t*_ and *m*_*t*_ be the values of these quantities at time *t*. They obey stochastic dynamics due to the noise, and are described by the the following coupled Langevin equations^7^,^14^,^16^:


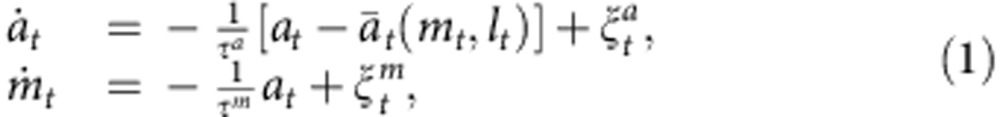


where 
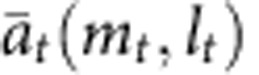
 is the stationary value of the kinase activity under the instantaneous values of the methylation level *m*_*t*_ and the ligand signal *l*_*t*_. In the case of *E. coli* chemotaxis, we can approximate 
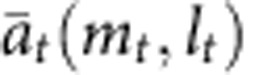
 as *αm*_*t*_−*βl*_*t*_, by linearizing it around the steady-state value^7^,^14^. 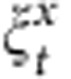 (*x* = *a*,*m*) is the white Gaussian noise with 
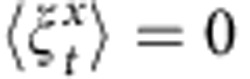
 and 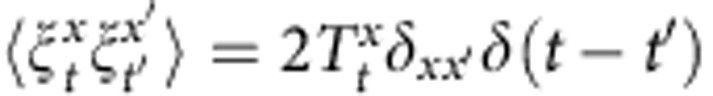, where 〈⋯〉 describes the ensemble average. 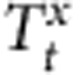 describes the intensity of the environmental noise at time *t*, which is not necessarily thermal inside cells. The noise intensity 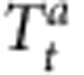 characterizes the ligand fluctuation. The time constants satisfy 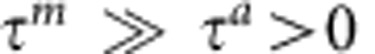, which implies that the relaxation of *a* to 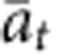 is much faster than that of *m*.

**Figure 2:**
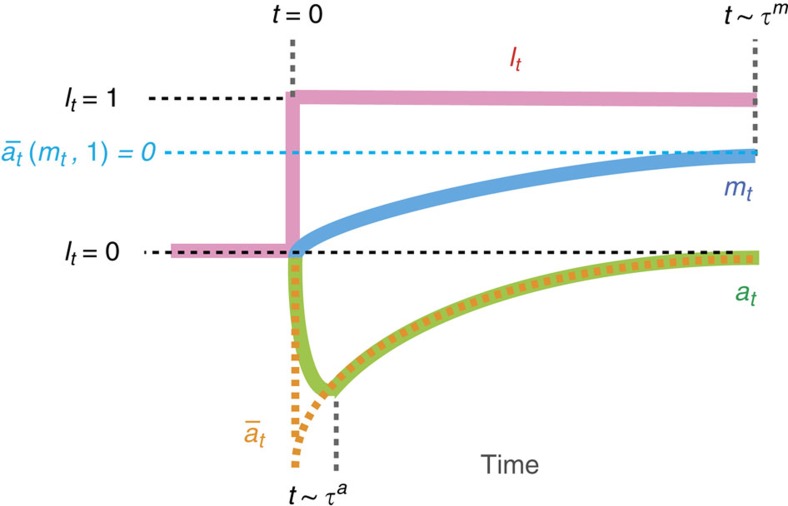
Typical dynamics of adaptation with the ensemble average. Suppose that *l*_*t*_ changes as a step function (red solid line). Then, *a*_*t*_ suddenly responds (green solid line), followed by the gradual response of *m*_*t*_ (blue solid line). The adaptation is achieved by the relaxation of *a*_*t*_ to 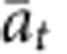 (orange dashed line). The methylation level *m*_*t*_ gradually changes to 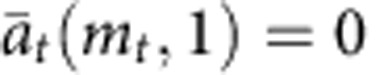 (blue dashed line).

The mechanism of adaptation in this model is as follows (Fig. 2; refs 14, 16). Suppose that the system is initially in a stationary state with *l*_*t*_ = 0 and 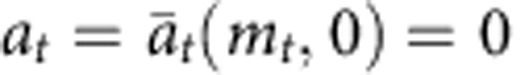 at time *t* < 0, and *l*_*t*_ suddenly changes from 0 to 1 at time *t* = 0 as a step function. Then, *a*_*t*_ rapidly equilibrates to 
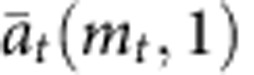
 so that the difference 
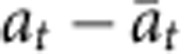
 becomes small. The difference 
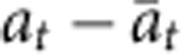
 plays an important role, which characterizes the level of adaptation. Next, *m*_*t*_ gradually changes to satisfy 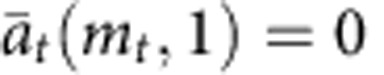, and thus *a*_*t*_ returns to 0, where 
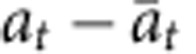
 remains small.

### Robustness against environmental noise

We introduce a key quantity that characterizes the robustness of adaptation, which is defined as the difference between the intensity of the ligand noise 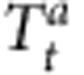 and the mean square error of the level of adaptation 
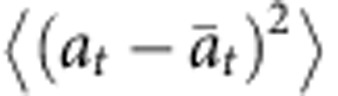
:


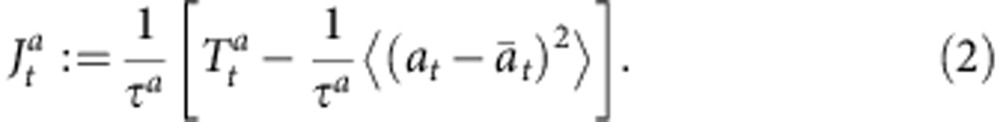


The larger 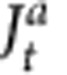 is, the more robust the signal transduction is against the environmental noise. In the case of thermodynamics, 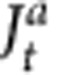 corresponds to the heat absorption in *a* and characterizes the violation of the fluctuation–dissipation theorem^28^. Since the environmental noise is not necessarily thermal in the present situation, 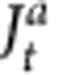 is not exactly the same as the heat, but is a biophysical quantity that characterizes the robustness of adaptation against the environmental noise.

### Information flow

We here discuss the quantitative definition of the transfer entropy^17^. The transfer entropy from *a* to *m* at time *t* is defined as the conditional mutual information between *a*_*t*_ and *m*_*t*+d*t*_ under the condition of *m*_*t*_:


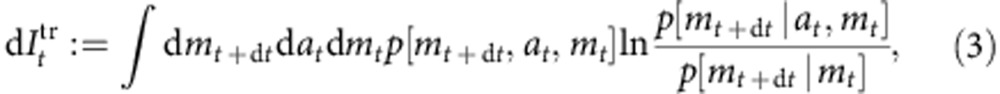


where *p*[*m*_*t*+d*t*_,*a*_*t*_,*m*_*t*_] is the joint probability distribution of (*m*_*t*+d*t*_,*a*_*t*_,*m*_*t*_), and *p*[*m*_*t*+d*t*_|*a*_*t*_,*m*_*t*_] is the probability distribution of *m*_*t*+d*t*_ under the condition of (*a*_*t*_,*m*_*t*_). The transfer entropy characterizes the directed information flow from *a* to *m* during an infinitesimal time interval d*t* (refs 17, 50), which quantifies a causal influence between them^51^,^52^. From the non-negativity of the conditional mutual information^23^, that of the transfer entropy follows: 
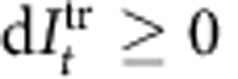
.

### Second law of information thermodynamics

**Figure 3:**
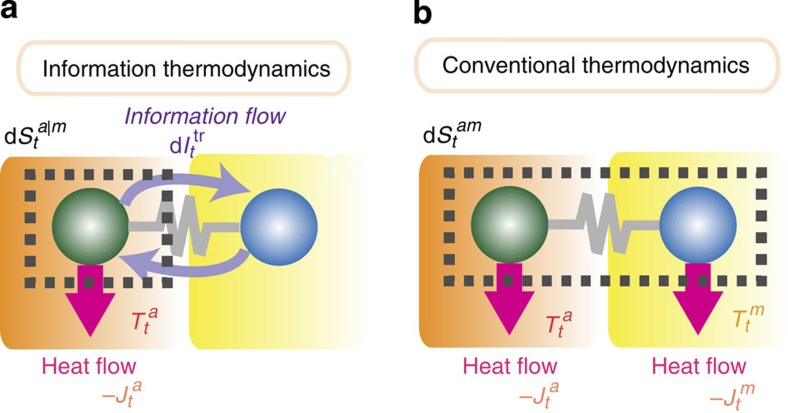
Schematics of information thermodynamics and conventional thermodynamics. A green (blue) circle indicates subsystem *a* (*m*) and a grey polygonal line indicates their interaction. (**a**) The second law of information thermodynamics characterizes the entropy change in a subsystem in terms of the information flow between the subsystem and the outside world (that is, 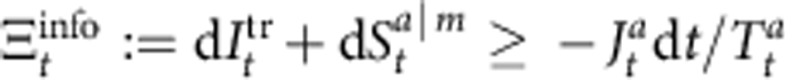). The information-thermodynamics picture concerns the entropy change inside the dashed square that only includes subsystem *a*. (**b**) the conventional second law of thermodynamics states that the entropy change in a subsystem is compensated for by the entropy change in the outside world (that is, 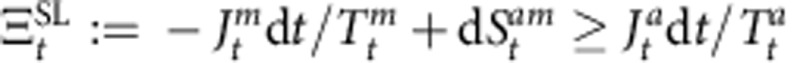). The conventional thermodynamics picture concerns the entropy change inside the dashed square, which includes the entire systems *a* and *m*. As explicitly shown in this paper, information thermodynamics gives a tighter bound of the robustness 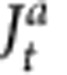 in the biochemical signal transduction of *E. coli* chemotaxis.

We now consider the second law of information thermodynamics, which characterizes the entropy change in a subsystem in terms of the information flow (Fig. 3). In the case of equation (1), the generalized second law is given as follows (see also Methods section):


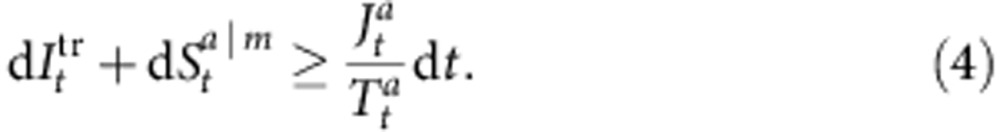


Here, 
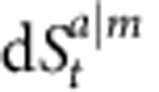
 is the conditional Shannon entropy change defined as 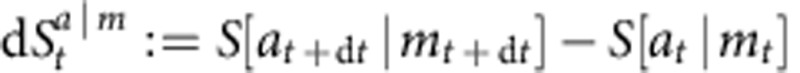 with 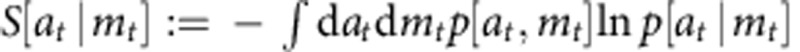, which vanishes in the stationary state. The transfer entropy d*I*_*t*_^tr^ on the left-hand side of equation (4) shows the significant role of the feedback loop, implying that the robustness of adaptation can be enhanced against the environmental noise by the feedback using information. This is analogous to the central feature of Maxwell's demon.

To further clarify the meaning of inequality (equation (4)), we focus on the case of the stationary state. If there was no feedback loop between *m* and *a*, then the second law reduces to 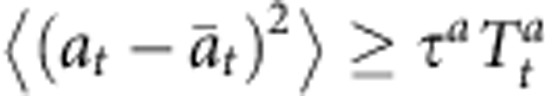, which, as naturally expected, implies that the fluctuation of the signal transduction is bounded by the intensity of the environmental noise. In contrast, in the presence of a feedback loop, 
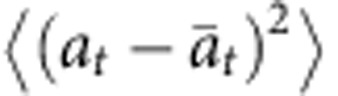
 can be smaller than 
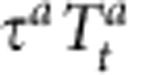
 owing to the transfer entropy 
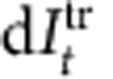
 in the feedback loop:


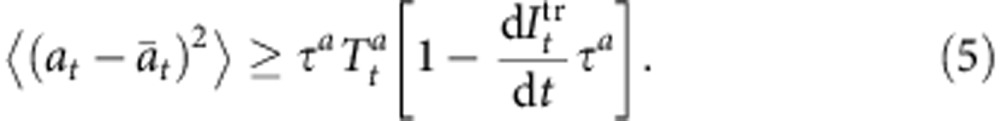


This inequality clarifies the role of the transfer entropy in biochemical signal transduction; the transfer entropy characterizes an upper bound of the robustness of the signal transduction in the biochemical network. The equality in equation (5) is achieved in the limit of *α* → 0 and *τ*^*a*^/*τ*^*m*^ → 0 for the linear case with 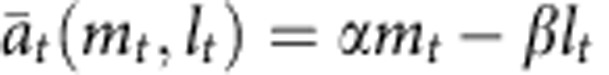 (Supplementary Note 1). The latter limit means that *a* relaxes infinitely fast and the process is quasi-static (that is, reversible) in terms of *a*. This is analogous to the fact that Maxwell's demon can achieve the maximum thermodynamics gain in reversible processes^35^. In general, the information-thermodynamic bound becomes tight if *α* and *τ*^*m*^/*τ*^*a*^ are both small. The realistic parameters of the bacterial chemotaxis are given by 
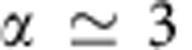
 and 
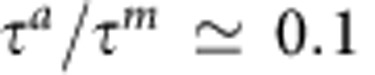
 (refs 7, 14, 16), and therefore the real adaptation process is accompanied by a finite amount of information-thermodynamics dissipation.

Our model of chemotaxis has the same mathematical structure as the feedback cooling of a colloidal particle by Maxwell's demon^36^,^38^,^42^,^47^, where the feedback cooling is analogous to the noise filtering in the sensory adaptation^49^. This analogy is a central idea of our study; the information-thermodynamic inequalities (equation (5) in our case) characterize the robustness of adaptation as well as the performance of feedback cooling.

### Numerical result

**Figure 4:**
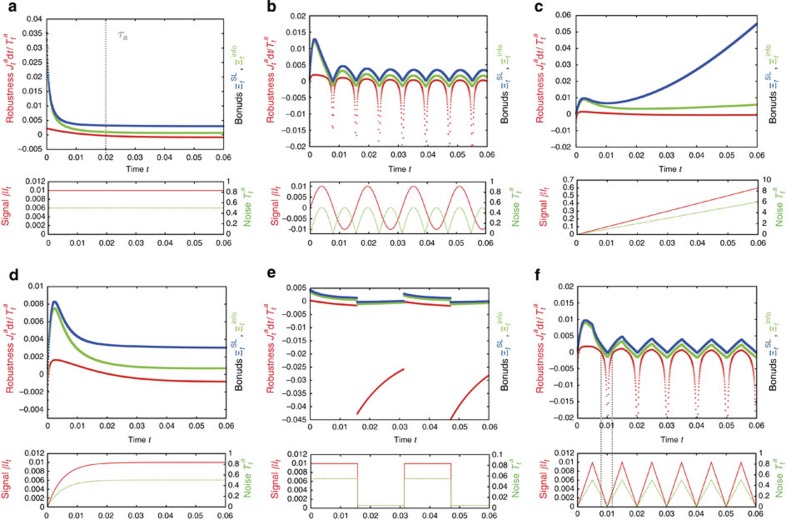
Numerical results of the information-thermodynamics bound on the robustness. We compare the robustness 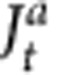 (red line), the information-thermodynamic bound 
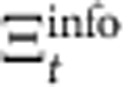
 (green line) and the conventional thermodynamic bound 
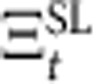
 (blue line). The initial condition is the stationary state with 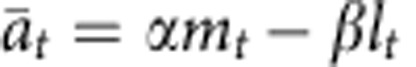, fixed ligand signal *βl*_*t*_ = 0, and noise intensity *T*^*a*^ = 0.005. We numerically confirmed that 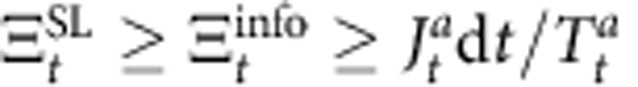 holds for the six transition processes. These results imply that, for the signal transduction model, the information-thermodynamic bound is tighter than the conventional thermodynamic bound. The parameters are chosen as *τ*^*a*^ = 0.02, *τ*^*m*^ = 0.2, *α* = 2.7 and 
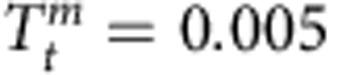
 to be consistent with the real parameters of *E. coli* bacterial chemotaxis 7,14,16. We discuss the six different types of input signals *βl*_*t*_ (red solid line) and noises 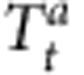 (green dashed line). (**a**) Step function: *βl*_*t*_ = 0.01 and 
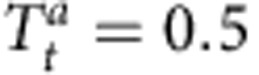
 for *t* > 0. (**b**) Sinusoidal function: *βl*_*t*_ = 0.01 sin(400*t*) and 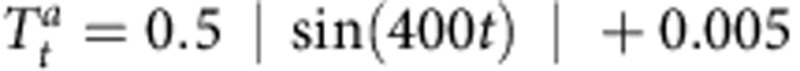 for *t* > 0. (**c**) Linear function: *βl*_*t*_ = 10*t* and 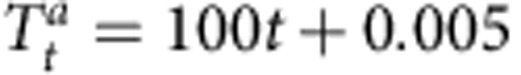 for *t* > 0. (**d**) Exponential decay: *βL*_*t*_ = 0.01[1−exp(−200*t*)] and 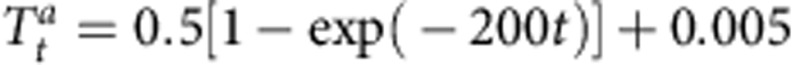 for *t* > 0. (**e**) Square wave: 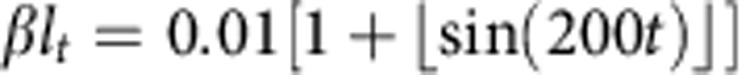 and 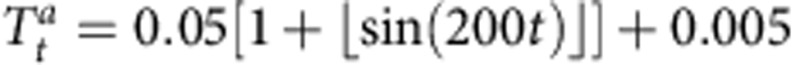 for *t* > 0, where 
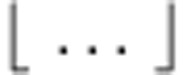
 denotes the floor function. (**f**) Triangle wave: 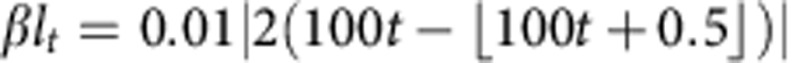 and 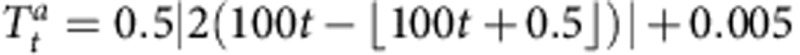 for *t* > 0.

We consider the second law (equation (4)) in non-stationary dynamics, and numerically demonstrate the power of this inequality. Figure 4 shows 
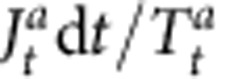
 and


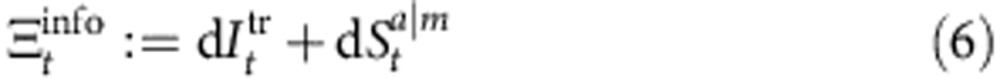


in six different types of dynamics of adaptation, where the ligand signal is given by a step function (Fig. 4a), a sinusoidal function (Fig. 4b), a linear function (Fig. 4c), an exponential decay (Fig. 4d), a square wave (Fig. 4e) and a triangle wave (Fig. 4f). These results confirm that 
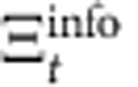
 gives a tight bound of 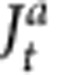, implying that the transfer entropy characterizes the robustness well. In Fig. 4b,f, the robustness 
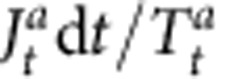
 is nearly equal to the information-thermodynamics bound 
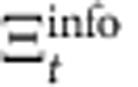
 when the signal and noise are decreasing or increasing rapidly (for example, 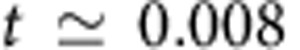 and *t* = 0.012 in Fig. 4f).

### Conventional second law of thermodynamics

For the purpose of comparison, we next consider another upper bound of the robustness, which is given by the conventional second law of thermodynamics without information. We define the heat absorption by *m* as 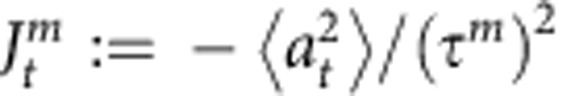, and the Shannon entropy change in the total system as 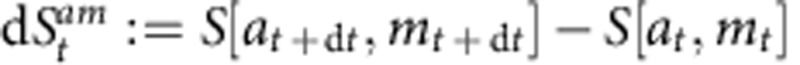 with 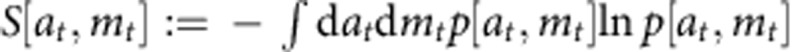, which vanishes in the stationary state. We can then show that


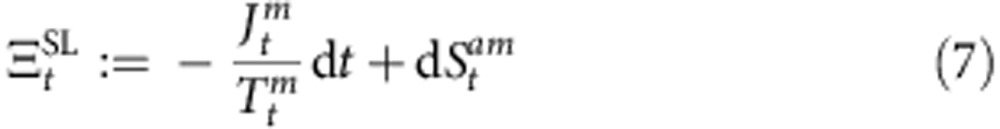


is an upper bound of 
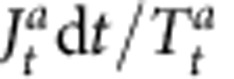
, as a straightforward consequence of the conventional second law of thermodynamics of the total system of *a* and *m* (refs 28, 29). The conventional second law implies that the dissipation in *m* should compensate for that in *a* (Fig. 3). Figure 4 shows 
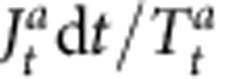
 along with 
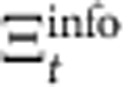
 and 
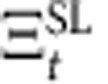
. Remarkably, information-thermodynamic bound 
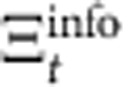
 gives a tighter bound of 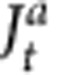 than the conventional thermodynamics bound 
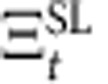
 such that


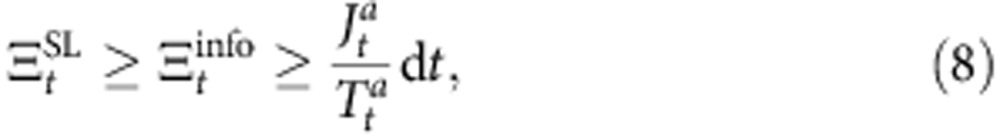


for every non-stationary dynamics shown in Fig. 4. Moreover, we can analytically show inequalities (equation (8)) in the stationary state (Supplementary Note 4).

To compare the information-thermodynamic bound and the conventional thermodynamics one more quantitatively, we introduce an information-thermodynamic figure of merit based on the inequalities (equation (8)):


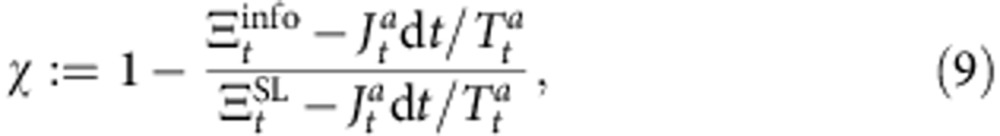


where the second term on the right-hand side is given by the ratio between the information-thermodynamic dissipation 
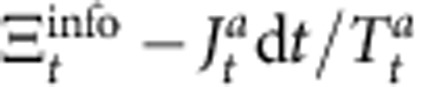
 and the entire thermodynamic dissipation 
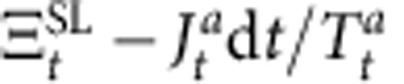
. This quantity satisfies 0 ≤ *χ* ≤ 1, and 
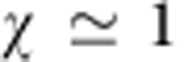
 (
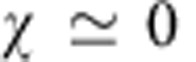
) means that information-thermodynamic bound is much tighter (a little tighter) compared with the conventional thermodynamic bound. We numerically calculated *χ* in the aforementioned six types of dynamics of adaptation (Supplementary Figs 1–6). In the case of a linear function (Supplementary Fig. 3), we found that χ increases in time *t* and approaches to 
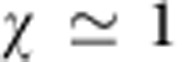
. In this case, the signal transduction of *E. coli* chemotaxis is highly dissipative as a thermodynamic engine, but efficient as an information transmission device.

### Comparison with Shannon's theory

**Figure 5:**
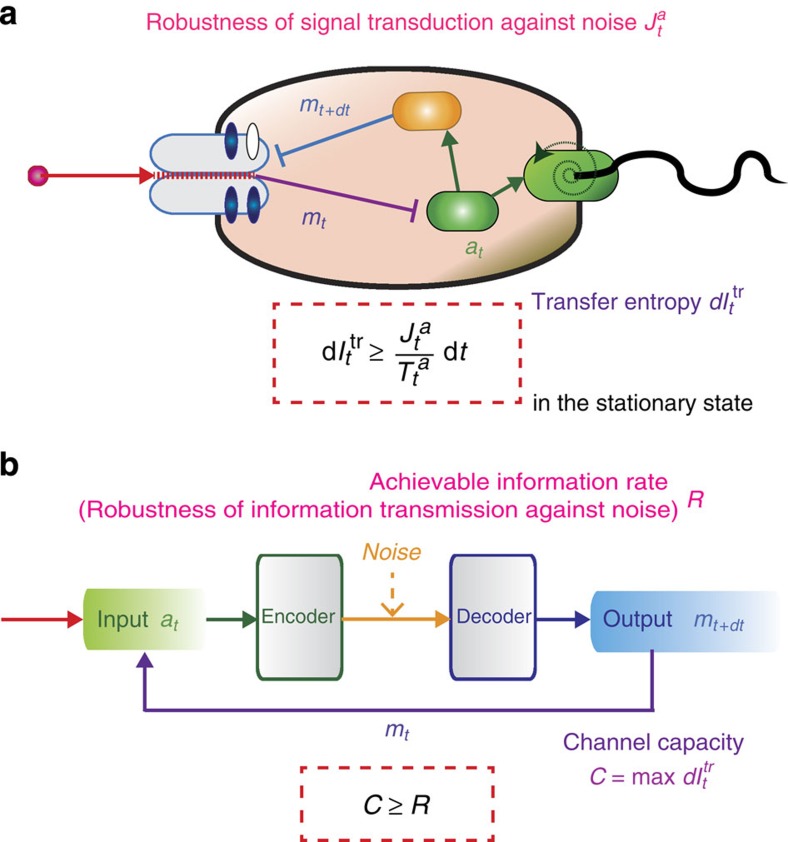
Analogy and difference between our approach and Shannon's information theory. (**a**) Information thermodynamics for biochemical signal transduction. The robustness 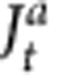 is bounded by the transfer entropy 
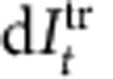
 in the stationary states, which is a consequence of the second law of information thermodynamics. (**b**) Information theory for artificial communication. The archivable information rate *R*, given by the redundancy of the channel coding, is bounded by the channel capacity 
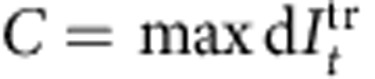
, which is a consequence of the Shannon's second theorem. If the noise is Gaussian as is the case for the *E. coli* chemotaxis, both of the transfer entropy and the channel capacity are given by the power-to-noise ratio 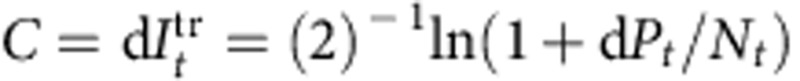, under the condition that the initial distribution is Gaussian (see Methods section).

We here discuss the similarity and the difference between our result and the Shannon's information theory (refs 22, 23; Fig. 5). The Shannon's second theorem (that is, the noisy-channel coding theorem) states that an upper bound of achievable information rate *R* is given by the channel capacity *C* such that *C* ≥ *R*. The channel capacity *C* is defined as the maximum value of the mutual information with finite power, where the mutual information can be replaced by the transfer entropy 
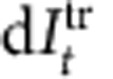
 in the presence of a feedback loop^21^. *R* describes how long bit sequence is needed for a channel coding to realize errorless communication through a noisy channel, where errorless means the coincidence between the input and output messages. Therefore, both of 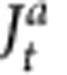 and *R* characterize the robustness information transmission against noise, and bounded by the transfer entropy 
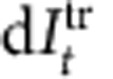
. In this sense, there exists an analogy between the second law of thermodynamics with information and the Shannon's second theorem. In the case of biochemical signal transduction, the information-thermodynamic approach is more relevant, because there is not any explicit channel coding inside cells. Moreover, while 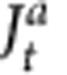 is an experimentally measurable quantity as mentioned below 28,29, *R* cannot be properly defined in the absence of any artificial channel coding 23. Therefore, 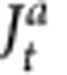 is an intrinsic quantity to characterize the robustness of the information transduction inside cells.

## Discussion

Our result can be experimentally validated by measuring the transfer entropy and thermodynamics quantities from the probability distribution of the amount of proteins in a biochemical system^5^,^6^,^9^,^10^,^46^,^47^,^48^,^49^. In fact, the transfer entropy d*I*_tr_ and thermodynamics quantities (that is, 
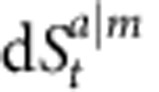
 and 
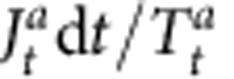
) can be obtained from the joint probability distribution of (*a*_*t*_,*m*_*t*_,*a*_*t*+d*t*_,*m*_*t*+d*t*_). The measurement of such a joint distribution would not be far from today's experimental technique in biophysics^5^,^6^,^9^,^10^,^53^,^54^,^55^,^56^. Experimental measurements of 
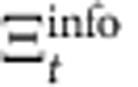
 and 
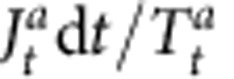
 would lead to a novel classification of signal transduction in terms of the thermodynamics cost of information transmission.

We note that, in ref. 16, the authors discussed that the entropy changes in two heat baths 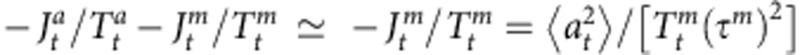 can be characterized by the accuracy of adaptation. In our study, we derived a bound for 
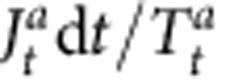
 that is regarded as the robustness of signal transduction against the environmental noise. These two results capture complementary aspects of adaptation processes: accuracy and robustness.

We also note that our theory of information thermodynamics^24^ can be generalized to a broad class of signal transduction networks, including a feedback loop with time delay.

## Methods

### The outline of the derivation of inequality (4)

We here show the outline of the derivation of the information-thermodynamic inequality (equation (4); see also Supplementary Note 2 for details). The heat dissipation 
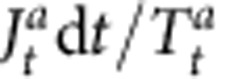
 is given by the ratio between forward and backward path probabilities as 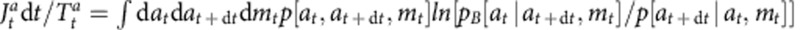 (refs 24, 28, 29), where the backward path probability 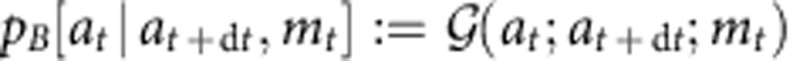 can be calculated from the forward path probability 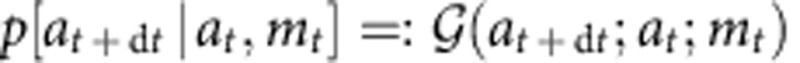. Thus, the difference 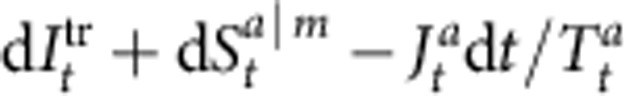 is given by the Kullback–Libler divergence^23^. From its non-negativity^23^, we have 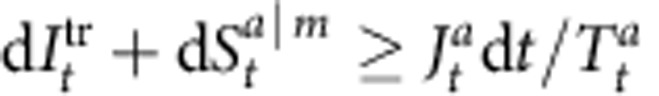. This inequality can be derived from the general inequality of information thermodynamics^24^ (see Supplementary Note 3 and Supplementary Fig. 7). As discussed in Supplementary Note 3, this inequality gives a weaker bound of the entropy production.

### The analytical expression of the transfer entropy

In the case of *E. coli* chemotaxis, we have 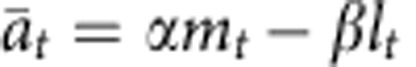, and equation (1) become linear. In this situation, if the initial distribution is Gaussian, we analytically obtain the transfer entropy up to the order of d*t* (Supplementary Note 4): 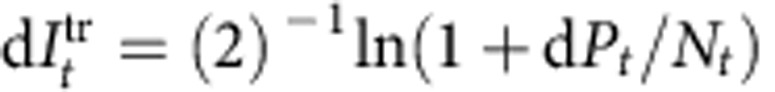, where 
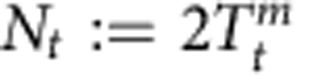
 describes the intensity of the environmental noise, and 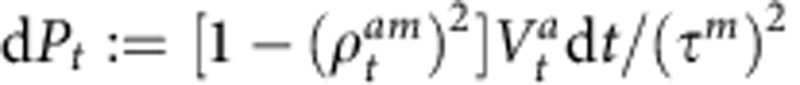 describes the intensity of the signal from *a* to *m* per unit time with 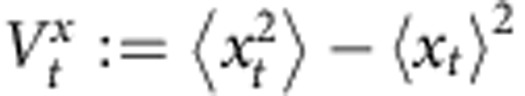, and 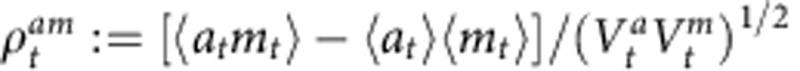. We note that d*I*_tr_ for the Gaussian case is greater than that of the non-Gaussian case, if 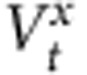 and 
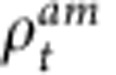
 are the same^23^. We also note that the above analytical expression of 
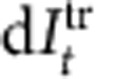
 is the same form as the Shannon–Hartley theorem^23^.

## Additional information

**How to cite this article:** Ito, S. and Sagawa, T. Maxwell's demon in biochemical signal transduction with feedback loop. *Nat. Commun.* 6:7498 doi: 10.1038/ncomms8498 (2015).

## Supplementary Material

Supplementary Information : Supplementary Figures 1-7, Supplementary Notes 1-4 and Supplementary References
