# Supplementary material for: Maxwell's demon in biochemical signal transduction with feedback loop
Source: Nat Commun. 2015 Jun 23;6:7498. doi: 10.1038/ncomms8498 (PMC4557369; doi:10.1038/ncomms8498)
Supplement: Supplementary Information — : Supplementary Figures 1-7, Supplementary Notes 1-4 and Supplementary References [file ncomms8498-s1.pdf]

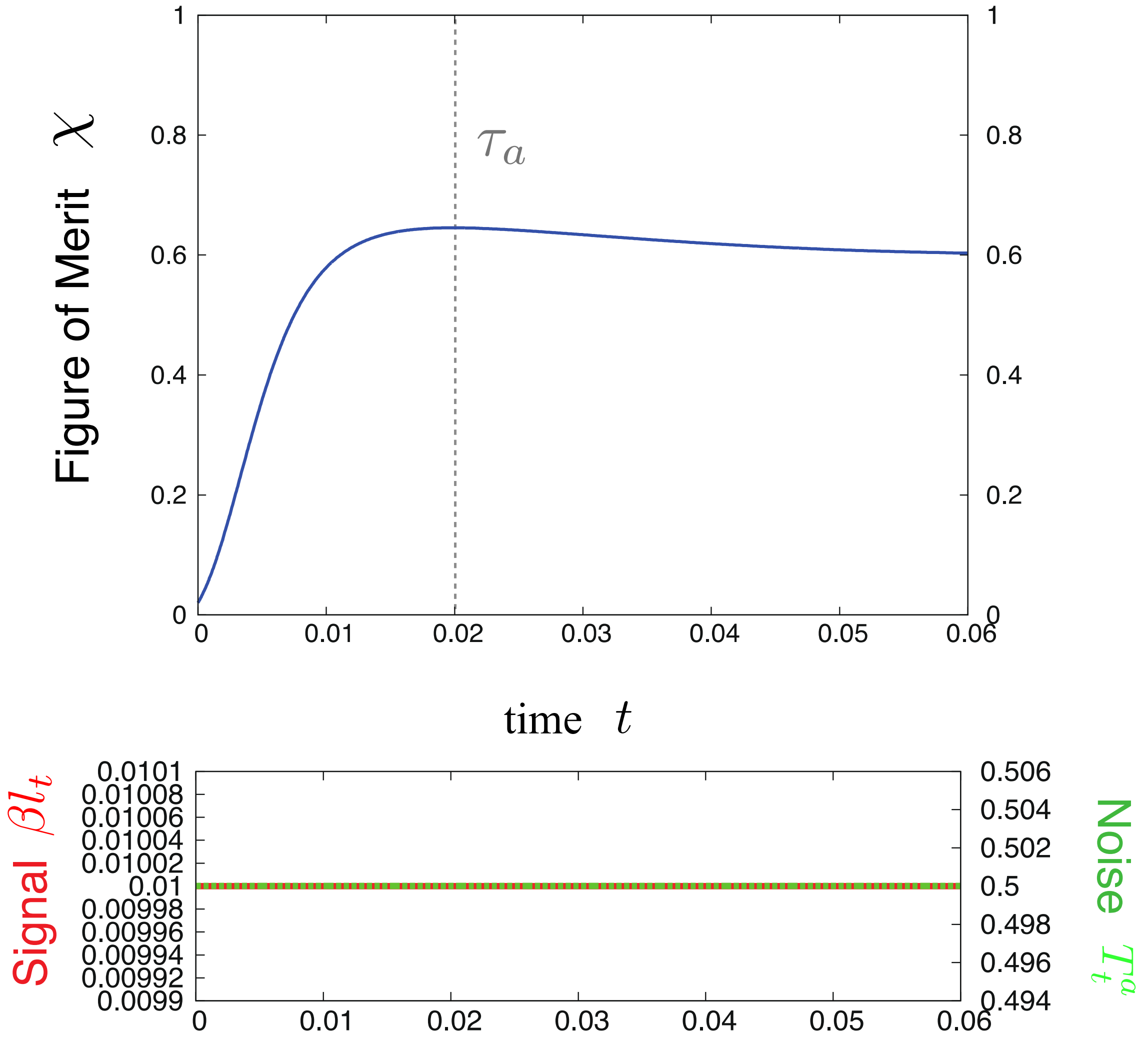

Supplementary Figure 1 | A figure of merit of information thermodynamics: Step function. The parameters are chosen as the same as in Fig. 4a in the main text.

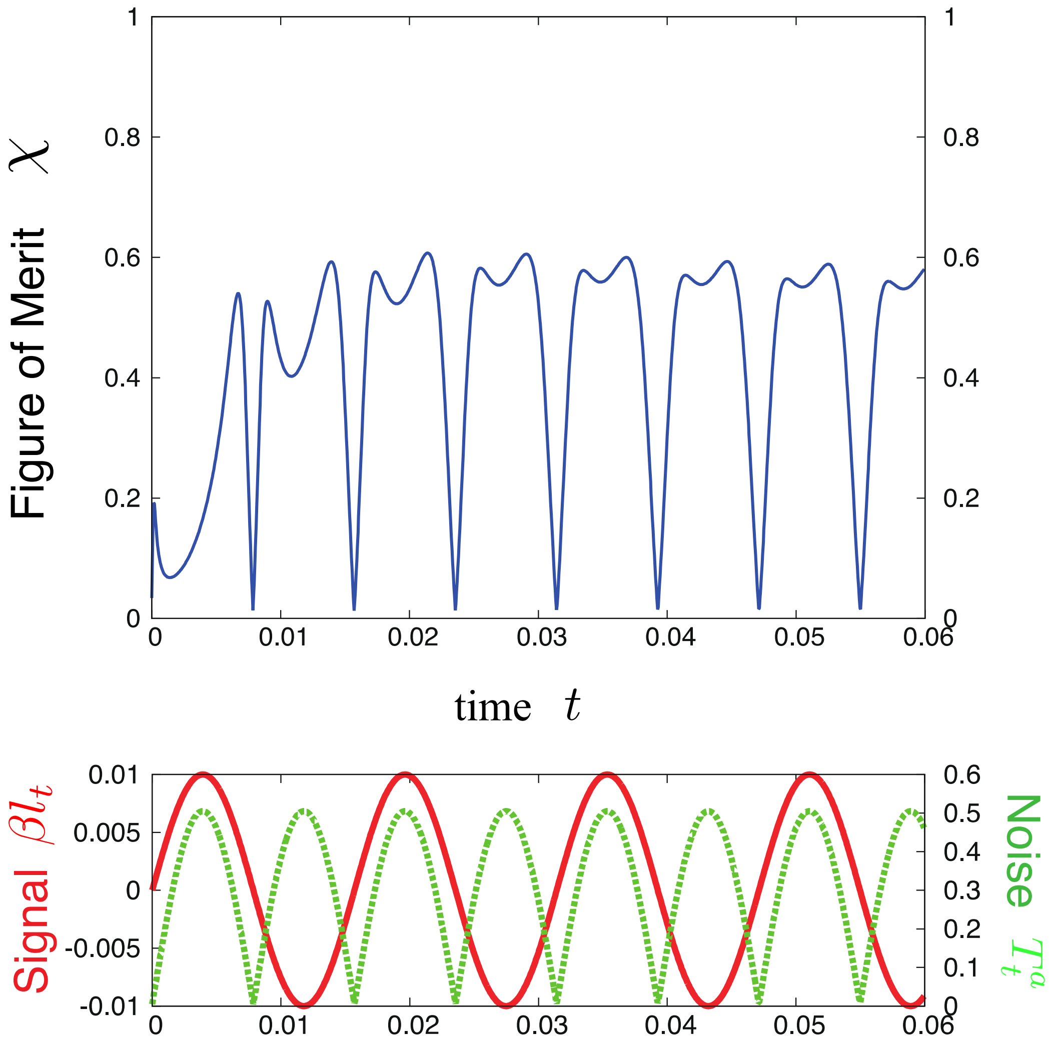

Supplementary Figure 2 | A figure of merit of information thermodynamics: Sinusoidal function. The parameters are chosen as the same as in Fig. 4b in the main text.

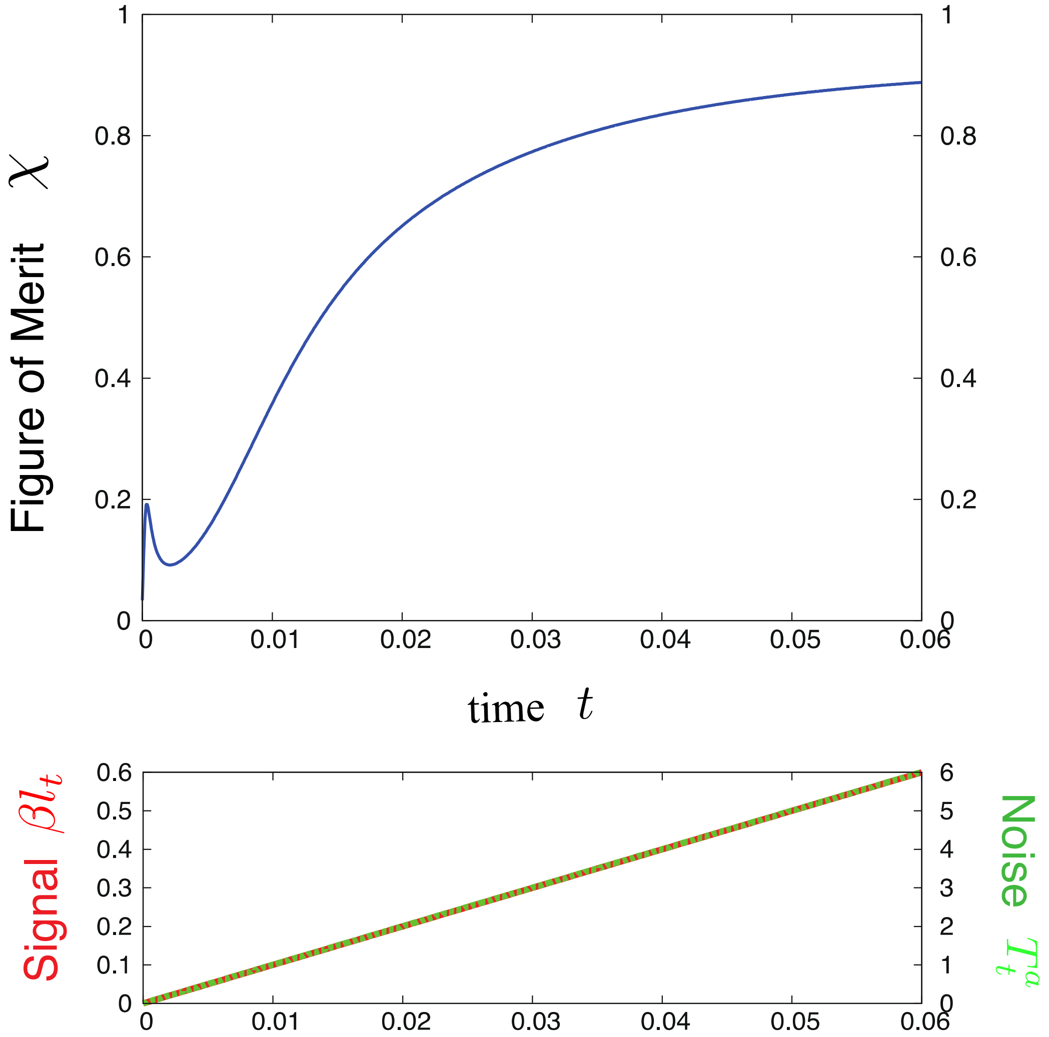

Supplementary Figure 3 | A figure of merit of information thermodynamics: Linear function. The parameters are chosen as the same as in Fig. 4c in the main text.

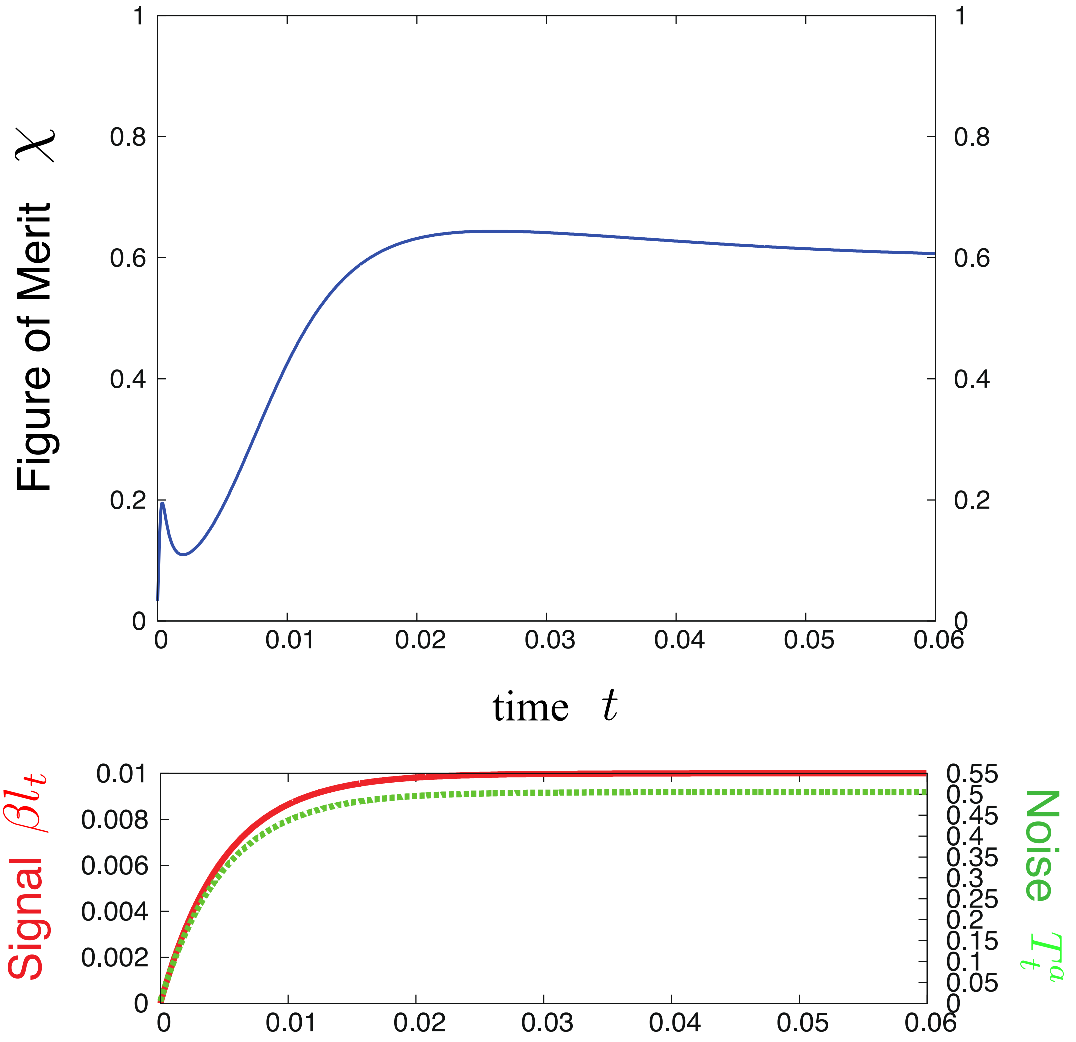

Supplementary Figure 4 | A figure of merit of information thermodynamics: Exponential decay. The parameters are chosen as the same as in Fig. 4d in the main text.

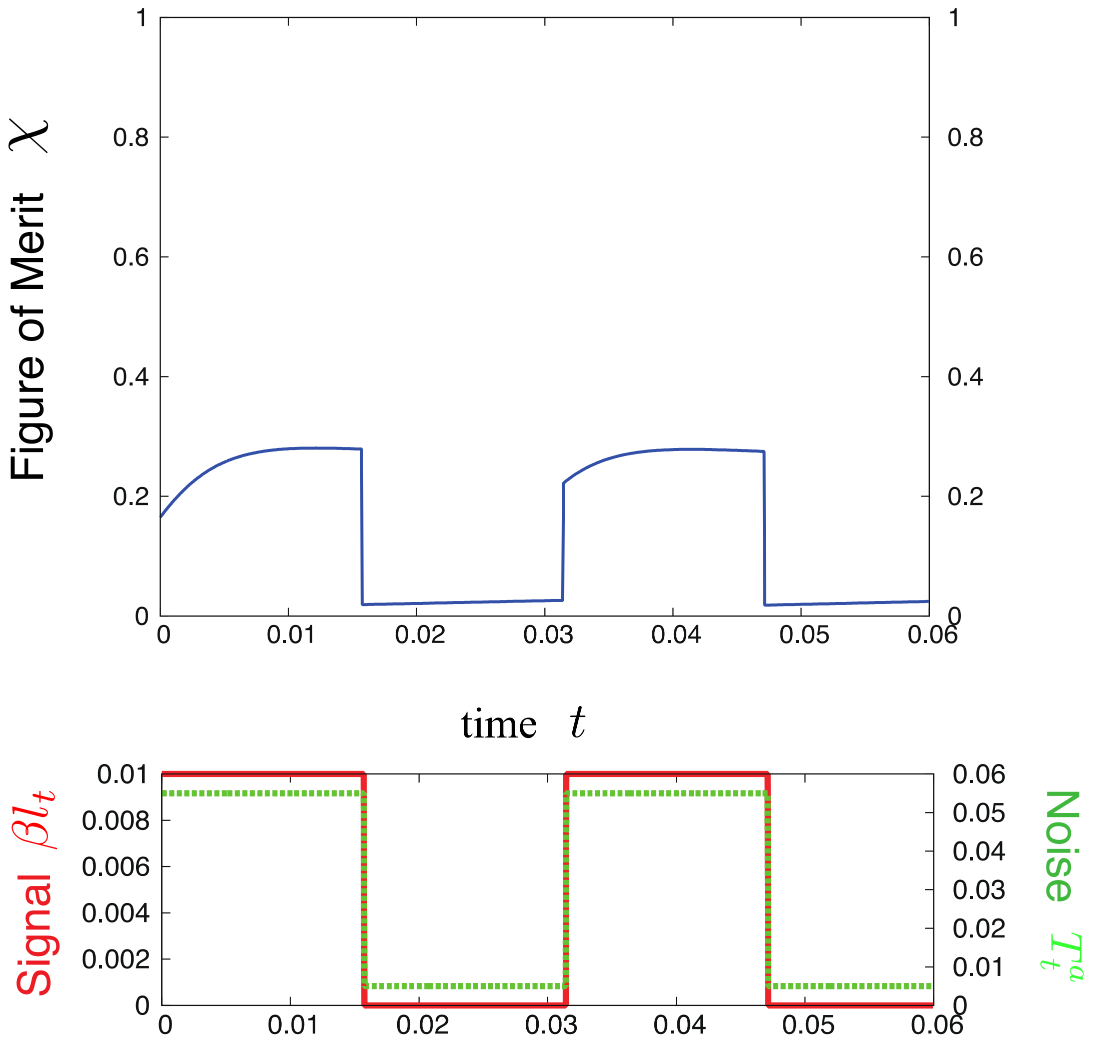

Supplementary Figure 5 | A figure of merit of information thermodynamics: Square wave. The parameters are chosen as the same as in Fig. 4e in the main text.

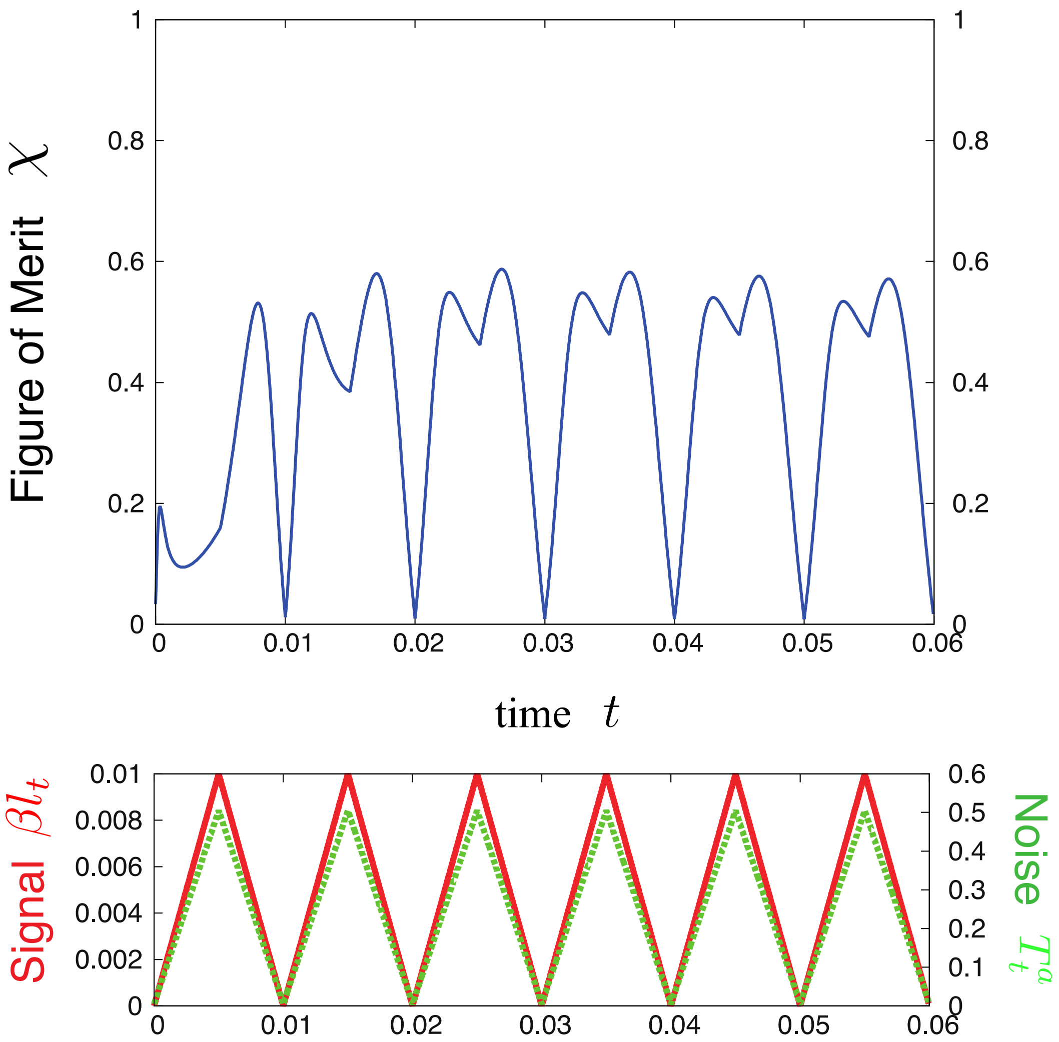

Supplementary Figure 6 | A figure of merit of information thermodynamics: Triangle wave. The parameters are chosen as the same as in Fig. 4f in the main text.

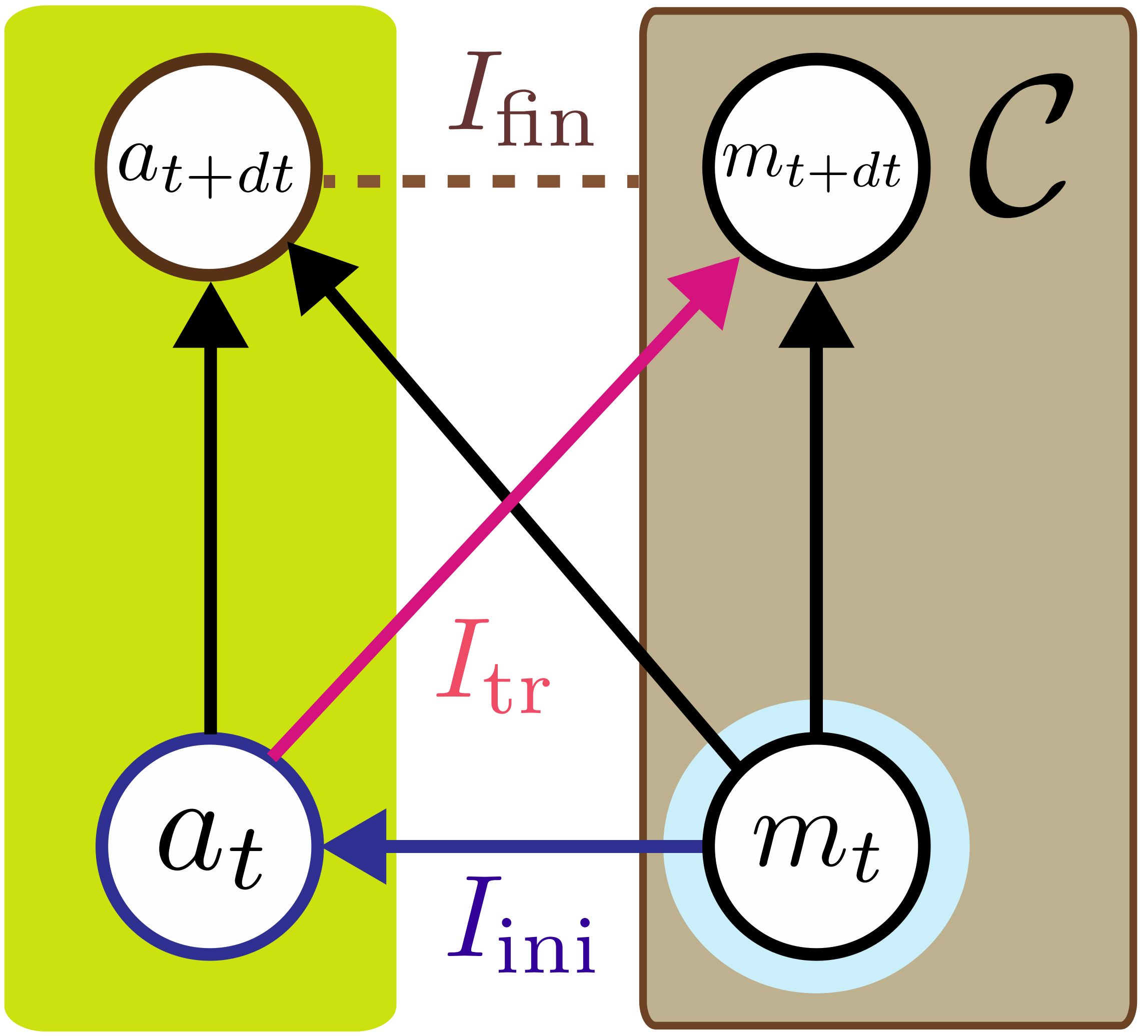

Supplementary Figure 7 | A Bayesian network corresponding to Eq. (22) in Supplementary note 3. This Bayesian network gives the joint probability Eq. (2), where a node represents a random variable and an edge represents a causal relationship. Due to a general framework of information thermodynamics[4], information of initial correlation I_{ini} is characterized by the mutual information between a_t and m_t, the information of final correlation I_{fin} is characterized by the mutual information between a_{t+dt} and $\{m_t, m_{t+dt}\}$, and the transfer entropy I_{tr} from the subsystem a to the other system $\mathcal{C}$ is characterized by the conditional mutual information between a_t and m_{t+dt} under the condition of m_t. These information quantities I_{ini}, I_{fin}, and I_{tr} give a lower bound of the entropy production in the subsystem a.

Supplementary note 1 | Explicit expression of the information-thermodynamic dissipation.

We consider the coupled Langevin equations (2) in the main text,

$$\dot{a}_t = -\frac{1}{\tau^a}[a_t - \bar{a}_t(m_t, l_t)] + \xi_t^a, \tag{1}$$

$$\dot{m}_t = -\frac{1}{\tau^m} a_t + \xi_t^m, \tag{2}$$

where ξ_t^x $(x = a, m)$ is a white Gaussian noise with the variance T_t^x: $\langle \xi_t^x \rangle = 0$, and $\langle \xi_t^x \xi_{t'}^{x'} \rangle = 2T_{t'}^{x'} \delta_{xx'} \delta(t - t')$. In the model of *E. coli* bacterial chemotaxis given by Eqs. (1) and (2) with $\bar{a}_t(m_t, l_t) = \alpha m_t - \beta l_t$, we can analytically calculate the information-thermodynamic dissipation in the stationary state:

$$dI_t^{\mathrm{tr}} - \frac{J_t^a}{T_t^a} dt$$

$$= \frac{[\langle a_t^2 \rangle - \langle a_t \rangle^2][1 - (\rho_t^{am})^2] dt}{4(\tau^m)^2 T_t^m} + \frac{dt}{\tau^a T_t^a}\left[\frac{1}{\tau^a}\langle (a_t - \bar{a}_t)^2 \rangle - T_t^a\right]. \tag{3}$$

When this quantity becomes zero, the equality in inequality (5) in the main text is achieved. With the linear approximation $\bar{a}_t(m_t, l_t) = \alpha m_t - \beta l_t$, we can explicitly calculate the stationary value of $\langle a_t \rangle$, $\langle m_t \rangle$, $\langle a_t^2 \rangle$, $\langle a_t m_t \rangle$ and $\langle m_t^2 \rangle$ as

$$\langle a_t \rangle_{\mathrm{SS}} = 0, \tag{4}$$

$$\langle m_t \rangle_{\mathrm{SS}} = \beta \alpha^{-1} l_t, \tag{5}$$

$$\langle a_t^2 \rangle_{\mathrm{SS}} = \alpha \tau^m T_t^m + \tau^a T_t^a, \tag{6}$$

$$\langle a_t m_t \rangle_{\mathrm{SS}} = \tau^m T_t^m, \tag{7}$$

$$\langle m_t^2 \rangle_{\mathrm{SS}} = (\beta \alpha^{-1} l_t)^2 + \alpha^{-1} \tau^m T_t^m + \tau^a \alpha^{-1} (\tau^m)^{-1} [\alpha \tau^m T_t^m + \tau^a T_t^a]. \tag{8}$$

The information-thermodynamic dissipation (3) then reduces to

$$dI_t^{\rm tr} - \frac{J_t^a}{T_t^a} dt$$

$$= dt[\alpha T_t^m + \tau^a (\tau^m)^{-1} T_t^a] \left[\frac{\alpha}{\tau^a T_t^a} + \frac{1-(\rho_t^{am})^2}{4\tau^m T_t^m} \right] \geq 0, \tag{9}$$

where the correlation coefficient $(\rho_t^{am})^2$ is given by

$$(\rho_t^{am})^2 = \frac{1}{[1+\tau^a(\tau^m)^{-1}[\alpha+\tau^a T_t^a(\tau^m T_t^m)^{-1}]][1+\tau^a T_t^a(\alpha \tau^m T_t^m)^{-1}]} \leq 1. \tag{10}$$

In the limit of $\alpha \to 0$ and $\tau^a/\tau^m \to 0$, the information-thermodynamic dissipation (3) can be zero, and the equality in Eq. (5) in the main text is achieved such that

$$dI_t^{\rm tr} = \frac{J_t^a}{T_t^a} dt = 0. \tag{11}$$

This corresponds to the situation where the feedback loop does not work ($\alpha \to 0$) and the information flow vanishes, and a relaxes infinitely fast ($\tau^a/\tau^m \to 0$).

Supplementary note 2 | Detailed derivation of the second law of information thermodynamics.

Here, we show the detailed derivation of the second law of information thermodynamics for Eqs. (1) and (2) [Eq. (4) in the main text]:

$$\Xi_t^{\rm info} := dI_t^{\rm tr} + dS_t^{a|m} \geq \frac{J_t^a}{T_t^a} dt, \tag{12}$$

where $dS_t^{a|m} := S[a_{t+dt}|m_{t+dt}] - S[a_t|m_t]$ is the conditional Shannon entropy change of a with $S[a_t|m_t] := -\int da_t\, dm_t p[a_t, m_t] \ln p[a_t|m_t]$, and $dI_t^{\rm tr}$ is the transfer entropy from a to m at time t:

$$dI_t^{\rm tr} := \int dm_{t+dt} da_t\, dm_t p[m_{t+dt}, a_t, m_t] \ln \frac{p[m_{t+dt}|a_t, m_t]}{p[m_{t+dt}|m_t]}, \tag{13}$$

The heat absorption[1] J_t^a is defined as the ensemble average of the Stratonovich product of the force $\xi_t^a - \dot{a}_t$ and the velocity $\dot{a}_t$ such that

$$J_t^a := \langle (\xi_t^a - \dot{a}_t) \circ \dot{a}_t \rangle, \tag{14}$$

The heat absorption J_t^a can be rewritten by Eq. (3) in the main text:

$$J_t^a = \langle (\xi_t^a - \dot{a}_t) \circ \dot{a}_t \rangle$$

$$= \frac{1}{\tau^a} \left[\langle (a_t - \bar{a}_t) \circ \xi_t^a \rangle - \frac{1}{\tau^a} \langle (a_t - \bar{a}_t)^2 \rangle \right]$$

$$= \frac{1}{\tau^a}\left[T_t^a - \frac{1}{\tau^a}\langle(a_t - \bar{a}_t)^2\rangle\right], \quad (15)$$

where we used the relation of the Stratonovich integral[1] $\langle f(a_t, m_t, l_t) \circ \xi_t^a\rangle = T_t^a\langle\partial_{a_t} f(a_t, m_t, l_t)\rangle$ for any function f.

From the detailed fluctuation theorem[2], $J_t^a dt/T_t^a$ can be rewritten as a ratio of the probability distribution. Let the backward path-probability $p_B[a_t|a_{t+dt}, m_t]$ be $p_B[a_t|a_{t+dt}, m_t] \coloneqq \mathcal{G}(a_t; a_{t+dt}; m_t)$, where $\mathcal{G}$ is given by the path-integral expression:

$$p[a_{t+dt}|a_t, m_t] = \mathcal{N} exp\left[-\frac{dt}{4T_t^a}\left(\frac{a_{t+dt} - a_t}{dt} + \frac{1}{\tau^a}(a_t - \bar{a}_t)\right)^2\right] \quad (16)$$

$$=: \mathcal{G}(a_{t+dt}; a_t; m_t). \quad (17)$$

$\mathcal{N}$ is the normalization constant, so that $\int da_{t+dt}\, \mathcal{G}(a_{t+dt}; a_t; m_t) = 1$ is satisfied. The backward path probability also satisfies the normalization condition $\int da_t\, p_B[a_t|a_{t+dt}, m_t] = \int da_t\, \mathcal{G}(a_t; a_{t+dt}; m_t) = 1$. Up to order dt, the entropy change in the heat bath with temperature T_t^a is calculated as

$$\frac{J_t^a}{T_t^a} dt \coloneqq \int da_{t+dt} da_t\, dm_t p[a_{t+dt}, a_t, m_t] \ln \frac{p_B[a_t|a_{t+dt}, m_t]}{p[a_{t+dt}|a_t, m_t]}, \quad (18)$$

which is well known as the detailed fluctuation theorem[2].

Because of the noise independence $\langle\xi_t^a \xi_{t'}^m\rangle = 0$, we have $p[a_{t+dt}, m_{t+dt}, a_t, m_t] = p[a_{t+dt}|a_t, m_t]p[m_{t+dt}|a_t, m_t]p[a_t, m_t]$. From Eqs. (13) and (18), the difference $\Xi_t^{\mathrm{info}} - J_t^a dt/\, T_t^a$ is calculated as

$$\Xi_t^{\mathrm{info}} - \frac{J_t^a}{T_t^a} dt = \left\langle \ln \frac{p[a_{t+dt}, m_{t+dt}, a_t, m_t]}{p[a_{t+dt}|m_{t+dt}]p_B[a_t|a_{t+dt}, m_t]p[m_{t+dt}, m_t]} \right\rangle. \quad (19)$$

The quantity $\mathcal{Q}[a_{t+dt}, m_{t+dt}, a_t, m_t] \coloneqq p[a_{t+dt}|m_{t+dt}]p_B[a_t|a_{t+dt}, m_t]p[m_{t+dt}, m_t]$ satisfies the normalization condition of the probability:

$$\int da_{t+dt} dm_{t+dt} da_t\, dm_t\, \mathcal{Q}[a_{t+dt}, m_{t+dt}, a_t, m_t] = 1. \quad (20)$$

Therefore, $\mathcal{Q}[a_{t+dt}, m_{t+dt}, a_t, m_t]$ can be interpreted as the probability distribution of $(a_{t+dt}, m_{t+dt}, a_t, m_t)$, and the difference $\Xi_t^{\mathrm{info}} - J_t^a dt/\, T_t^a$ is rewritten as the Kullback-Libler divergence $D_{KL}(p||\mathcal{Q})$[3]:

$$\Xi_t^{\mathrm{info}} - \frac{J_t^a}{T_t^a} dt$$

$$= \int da_{t+dt} dm_{t+dt} da_t \, dm_t p[a_{t+dt}, m_{t+dt}, a_t, m_t] \ln \frac{p[a_{t+dt}, m_{t+dt}, a_t, m_t]}{\mathcal{Q}[a_{t+dt}, m_{t+dt}, a_t, m_t]}$$

$$:= D_{KL}(p||\mathcal{Q}). \tag{21}$$

From the non-negativity of the Kullback-Leibler divergence[3] [i.e., $D_{KL}(p||\mathcal{Q}) \geq 0$], we obtain Eq. (12).

Supplementary note 3 | Relationship between information thermodynamics for two-dimensional Markov process and that in [S. Ito and T. Sagawa, Phys. Rev. Lett. 111, 180503 (2013)].

In our previous paper[4], we have derived a general framework of information thermodynamics and discussed information thermodynamics for the coupled Langevin equations. We here give another application of the general result in Ref. 4 to two-dimensional Markov processes such as the coupled Langevin equations (1) and (2). Here, we show that the general result in Ref. 4 is tighter than the information-thermodynamic inequality (12).

We first consider the path probability of a single time step from (a_t, m_t), to (a_{t+dt}, m_{t+dt}). Due to the Markov property, the joint probability $p[a_{t+dt}, m_{t+dt}, a_t, m_t]$ is given by

$$p[a_{t+dt}, m_{t+dt}, a_t, m_t] = p[a_{t+dt}|a_t, m_t] p[m_{t+dt}|a_t, m_t] p[a_t|m_t] p[m_t], \tag{22}$$

where the independency of the noise (i.e., $p[a_{t+dt}, m_{t+dt}|a_t, m_t] = p[a_{t+dt}|a_t, m_t] p[m_{t+dt}|a_t, m_t]$) is assumed.

We next consider a Bayesian network, which represents the stochastic process of Eq. (22) (see Supplementary Fig. 7). This Bayesian network is given by the parents (denoted as “pa”) of the random variables: $\mathrm{pa}(a_t) = m_t$, $\mathrm{pa}(m_t) = \emptyset$, $\mathrm{pa}(a_{t+dt}) = \{a_t, m_t\}$ and $\mathrm{pa}(m_{t+dt}) = \{a_t, m_t\}$. The stochastic process of Eq. (22) is given by $p[a_{t+dt}, m_{t+dt}, a_t, m_t] = p[a_{t+dt}|\mathrm{pa}(a_{t+dt})] p[m_{t+dt}|\mathrm{pa}(m_{t+dt})] p[a_t|\mathrm{pa}(a_t)] p[m_t|\mathrm{pa}(m_t)]$. This Bayesian network shows a single time step of the Markovian dynamics from time t to time $t + dt$.

Let stochastic mutual information be $I[\mathcal{A}_1 : \mathcal{A}_2] \coloneqq \ln p[\mathcal{A}_1, \mathcal{A}_2] - \ln p[\mathcal{A}_1] - \ln p[\mathcal{A}_2]$, and stochastic conditional mutual information be $I[\mathcal{A}_1 : \mathcal{A}_2 | \mathcal{A}_3] \coloneqq \ln p[\mathcal{A}_1, \mathcal{A}_2 | \mathcal{A}_3] - \ln p[\mathcal{A}_1 | \mathcal{A}_3] - \ln p[\mathcal{A}_2 | \mathcal{A}_3]$, where $\mathcal{A}_1$, $\mathcal{A}_2$

and $\mathcal{A}_3$ are any set of random variables. From the argument in Ref. 4, the bound of the entropy production for the subsystem a is given by an informational quantity Θ, which corresponds to the Bayesian network shown in Supplementary Fig. 7:

$$\Theta \coloneqq I_{\mathrm{fin}} - I_{\mathrm{ini}} - \sum_{l=1}^{2} I_{\mathrm{tr}}^{l}, \tag{23}$$

$$\begin{aligned} I_{\mathrm{fin}} &= I[x_2 \colon \mathcal{C}] \\ &= I[a_{t+dt} \colon \{m_t, m_{t+dt}\}], \end{aligned} \tag{24}$$

$$\begin{aligned} I_{\mathrm{ini}} &= I[x_1 \colon \mathrm{pa}(x_1)] \\ &= I[a_t \colon m_t], \end{aligned} \tag{25}$$

$$\begin{aligned} I_{\mathrm{tr}}^{1} &= I[c_1 \colon \mathrm{pa}_X(c_1)] \\ &= 0, \end{aligned} \tag{26}$$

$$\begin{aligned} I_{\mathrm{tr}}^{2} &= I[c_2 \colon \mathrm{pa}_X(c_2) | c_1] \\ &= I[m_{t+dt} \colon a_t | m_t], \end{aligned} \tag{27}$$

where we set $X \colon = \{x_1 = a_t, x_2 = a_{t+dt}\}$, $\mathcal{C} \colon = \{c_1 = m_t, c_2 = m_{t+dt}\}$, $\mathrm{pa}_X(m_t) \coloneqq \mathrm{pa}(m_t) \cap X = \emptyset$, and $\mathrm{pa}_X(m_{t+dt}) \coloneqq \mathrm{pa}(m_{t+dt}) \cap X = a_t$. Let the entropy production in the subsystem during the infinitesimal time step be $\sigma_t \coloneqq \ln \mathrm{p}(a_t) - \ln \mathrm{p}(a_{t+dt}) + \Delta s_t^{\mathrm{bath}}$, where $\Delta s_t^{\mathrm{bath}}$ is the entropy change in the heat baths. Again from the argument in Ref. 4, we have inequality $\langle \sigma \rangle \geq \langle \Theta \rangle$, where

$$\langle \Theta \rangle = \langle I[a_{t+dt} \colon \{m_t, m_{t+dt}\}] \rangle - \langle I[a_t \colon \{m_t, m_{t+dt}\}] \rangle \tag{28}$$

$$= I_{t+dt}^{am} - I_t^{am} + dI_t^{\mathrm{Btr}} - dI_t^{\mathrm{tr}}. \tag{29}$$

$I_t^{am} \coloneqq \langle I[a_t \colon m_t] \rangle$ is the mutual information between a and m at time t, $dI_t^{\mathrm{tr}} \coloneqq \langle \ln p[m_{t+dt} | a_t, m_t] \rangle - \langle \ln p[m_{t+dt} | m_t] \rangle$ is the transfer entropy from a to m at time t, and dI_t^{Btr} is defined as the conditional mutual information $dI_t^{\mathrm{Btr}} = \langle I[m_t \colon a_{t+dt} | m_{t+dt}] \rangle$. We note that Eq. (28) is consistent with information flow in several papers[5-8].

For the two-dimensional Langevin system Eqs. (1) and (2), the ensemble average of the entropy production for the subsystem $\langle \sigma \rangle$ can be rewritten by the heat absorption J_t^a, $\langle \sigma \rangle = -J_t^a dt / T_t^a + \langle \ln p[a_t] \rangle - \langle \ln p[a_{t+dt}] \rangle$ with $\langle \Delta s_t^{\mathrm{bath}} \rangle = -J_t^a dt / T_t^a$. From $\langle \sigma \rangle \geq \langle \Theta \rangle$, we have the following inequality:

$$\frac{J_t^a}{T_t^a} dt \leq -dI_t^{\mathrm{Btr}} + dI_t^{\mathrm{tr}} + dS_t^{a|m} \tag{30}$$

where we used Eq. (29) and identity $dS_t^{a|m} = \langle \ln p[a_t] \rangle - \langle \ln p[a_{t+dt}] \rangle - I_{t+dt}^{am} + I_t^{am}$. Because of the non-negativity of the mutual information[3] [i.e., $dI_t^{\mathrm{Btr}} \geq 0$], we have inequality (12) [Eq. (4) in the main text]:

$$\frac{J_t^a}{T_t^a} dt \leq -dI_t^{\mathrm{Btr}} + dI_t^{\mathrm{tr}} + dS_t^{a|m} \tag{31}$$

$$\leq dI_t^{\mathrm{tr}} + dS_t^{a|m}. \tag{32}$$

The conditional mutual information dI_t^{Btr} would be important as well as the transfer entropy dI_t^{tr}, because the bound including dI_t^{Btr} [Eq. (31)] is tighter than the bound without dI_t^{Btr} [Eq. (32)]. However, in the main text, we only focus on the role of the transfer entropy dI_t^{tr} for the sake of simplicity, by applying the weaker inequality (32).

Supplementary note 4 | Analytical calculation of the transfer entropy for the coupled linear Langevin system.

We derive the analytical expression of the transfer entropy for the coupled linear Langevin system:

$$\begin{gathered} \dot{x}_t^1 = \textstyle\sum_j^2 \mu_t^{1j} x_t^j + f_t^1 + \xi_t^1, \\ \dot{x}_t^2 = \textstyle\sum_j^2 \mu_t^{2j} x_t^j + f_t^2 + \xi_t^2, \\ \langle \xi_t^i \xi_{t'}^j \rangle = 2T_t^i \delta_{ij} \delta(t-t'), \\ \langle \xi_t^i \rangle = 0, \end{gathered} \tag{33}$$

where i,j=1,2, f_t^i and μ_t^{ij} are the time-dependent constants, T_t^i is time-dependent variance of the white Gaussian noise ξ_t^i, and $\langle \ldots \rangle$ denotes the ensemble average. In the main text, we considered the model of the *E. coli* bacterial chemotaxis given by Eqs. (1) and (2) with $\bar{a}_t(m_t, l_t) = \alpha m_t - \beta l_t$. To compare Eqs. (1) and (2), we set $\{x_t^1, x_t^2\} = \{a_t, m_t\}$, $\mu_t^{11} = -1/\tau^a$, $\mu_t^{12} = \alpha/\tau^a$, $f_t^1 = -\beta l_t/\tau^a$, $\mu_t^{21} = -1/\tau^m$, $\mu_t^{22} = 0$, $f_t^2 = 0$, $T_t^1 = T_t^a$, and $T_t^2 = T_t^m$. The transfer entropy from the target system x^1 to the other system x^2 at time t is defined as

$dI_t^{\mathrm{tr}} \coloneqq \langle \ln p[x_{t+dt}^2 | x_t^1, x_t^2] \rangle - \langle \ln p[x_{t+dt}^2 | x_t^2] \rangle$.

Here, we analytically calculate the transfer entropy for the case that the joint probability $p[x_t^1, x_t^2]$ is a Gaussian distribution:

$$p[x_t^1, x_t^2] = \frac{1}{2\pi\sqrt{\det\Sigma_t}} \exp\left[-\sum_{ij} \frac{1}{2} \bar{x}_t^i G_t^{ij} \bar{x}_t^j \right], \tag{34}$$

where Σ_t is the covariant matrix $\Sigma_t^{ij} = \langle \bar{x}_t^i \bar{x}_t^j \rangle$, and $\bar{x}_t^i = x_t^i - \langle x_t^i \rangle$. The inverse matrix $G_t = (\Sigma_t)^{-1}$ satisfies $\sum_j G_t^{ij} \Sigma_t^{jl} = \delta_{il}$ and $G_t^{ij} = G_t^{ji}$. The joint distribution $p[x_t^2]$ is given by the Gaussian probability:

$$p[x_t^2] = \frac{1}{\sqrt{2\pi \Sigma_t^{22}}} \exp\left[-\frac{1}{2} (\Sigma_t^{22})^{-1} (\bar{x}_t^2)^2 \right], \tag{35}$$

We consider the path-integral expression of the Langevin equations (33). The conditional probability $p[x_{t+dt}^2 | x_t^1, x_t^2]$ is given by

$$\begin{aligned} p[x_{t+dt}^2 | x_t^1, x_t^2] &= \mathcal{N} \exp\left[-\frac{dt}{4T_t^2} \left(\frac{x_{t+dt}^2 - x_t^2}{dt} - \sum_j^2 \mu_t^{2j} x_t^j - f_t^2 \right)^2 \right] \\ &= \mathcal{N} \exp\left[-\frac{dt}{4T_t^2} (F_t^2 - \mu_t^{21} \bar{x}_t^1)^2 \right], \end{aligned} \tag{36}$$

where $\mathcal{N}$ is the normalization constant with $\int dx_{t+dt}^2 p[x_{t+dt}^2 | x_t^1, x_t^2] = 1$.

For the simplicity of notation, we set $F_t^2 := (x_{t+dt}^2 - x_t^2)/dt - \mu_t^{22} \langle x_t^1 \rangle - \mu_t^{22} x_t^2 - f_t^2$.

From Eqs. (34) and (36), we have the joint distribution $p[x_{t+dt}^2, x_t^2]$ as

$$\begin{aligned} &p[x_{t+dt}^2, x_t^2] \\ &= \int dx_t^1 p[x_{t+dt}^2 | x_t^1, x_t^2] \, p[x_t^1, x_t^2] \\ &= \frac{\mathcal{N}}{\sqrt{4\pi \det\Sigma_t \left(\frac{dt}{4T_t^2} (\mu_t^{21})^2 + \frac{G_t^{11}}{2} \right)}} \exp\left[-\frac{dt}{4T_t^2} (F_t^2)^2 - \frac{1}{2} G_t^{22} (\bar{x}_t^2)^2 \right. \\ &\left. + \frac{\left(G_t^{12} \bar{x}_t^2 - \frac{\mu_t^{21} F_t^2}{2T_t^2} dt \right)^2}{4 \left(\frac{dt}{4T_t^2} (\mu_t^{21})^2 + \frac{G_t^{11}}{2} \right)} \right]. \end{aligned} \tag{37}$$

From Eqs. (35), (36), and (37), we obtain the analytical expression of the transfer entropy dI_t^{tr} up to the order of dt:

$$\begin{aligned} &dI_t^{\mathrm{tr}} \\ &= \langle \ln p[x_{t+dt}^2 | x_t^1, x_t^2] \rangle + \langle \ln p[x_t^2] \rangle - \langle \ln p[x_{t+dt}^2, x_t^2] \rangle \end{aligned}$$

$$= -\frac{dt}{4T_t^2}\langle(F_t^2 - \mu_t^{21}\bar{x}_t^1)^2\rangle - \frac{1}{2}\ln[2\pi\Sigma_t^{22}] - \frac{1}{2}(\Sigma_t^{22})^{-1}\langle(\bar{x}_t^2)^2\rangle$$

$$+\frac{1}{2}\ln\left[4\pi\det\Sigma_t\left(\frac{dt}{4T_t^2}(\mu_t^{21})^2 + \frac{G_t^{11}}{2}\right)\right] + \frac{dt}{4T_t^2}\langle(F_t^2)^2\rangle + \frac{1}{2}G_t^{22}\langle(\bar{x}_t^2)^2\rangle$$

$$+\frac{\left\langle\left(G_t^{12}\bar{x}_t^2 - \frac{\mu_t^{21}F_t^2}{2T_t^2}dt\right)^2\right\rangle}{4\left(\frac{dt}{4T_t^2}(\mu_t^{21})^2 + \frac{G_t^{11}}{2}\right)}$$

$$= \frac{\mu_t^{21}dt}{2T_t^2}\langle F_t^2\bar{x}_t^1\rangle - \frac{dt}{4T_t^2}(\mu_t^{21})^2\Sigma_t^{11} - \frac{1}{2}\ln[2\pi\Sigma_t^{22}] - \frac{1}{2} + \frac{(\mu_t^{21})^2 dt}{4G_t^{11}T_t^2}$$

$$+\frac{1}{2}G_t^{22}\Sigma_t^{22} - \frac{(G_t^{12})^2\Sigma_t^{22}}{2G_t^{11}}\left[1 - \frac{(\mu_t^{21})^2 dt}{2G_t^{11}T_t^2}\right] + \frac{\mu_t^{21}dt}{2G_t^{11}T_t^2}G_t^{12}\langle F_t^2\bar{x}_t^2\rangle$$

$$-\frac{(\mu_t^{21})^2 dt}{4G_t^{11}T_t^2} + \mathcal{O}(dt^2)$$

$$= \frac{\mu_t^{21}dt}{2T_t^2}\langle F_t^2\bar{x}_t^1\rangle + \frac{\mu_t^{21}dt}{2G_t^{11}T_t^2}G_t^{12}\langle F_t^2\bar{x}_t^2\rangle - \frac{(\mu_t^{21})^2 dt}{4G_t^{11}T_t^2} + \mathcal{O}(dt^2)$$

$$= \frac{(\mu_t^{21})^2}{4T_t^2}\frac{\det\Sigma_t}{\Sigma_t^{22}}dt + \mathcal{O}(dt^2)$$

$$= \frac{1}{2}\ln\left(1 + \frac{dP_t}{N_t}\right) + \mathcal{O}(dt^2), \tag{38}$$

where we define $dP_t := (\mu_t^{21})^2(\det\Sigma_t)dt/(\Sigma_t^{22})$, and $N_t = 2T_t^2$. In this calculation, we used $G_t^{ij} = G_t^{ji}$, $\Sigma_t^{ij} = \Sigma_t^{ji}$, $G_t^{i1}\Sigma_t^{1l} + G_t^{i2}\Sigma_t^{2l} = \delta_{il}$, $\langle(F_t^2)^2\rangle dt^2 = 2T_t^2 dt + \mathcal{O}(dt^2)$, $\langle F_t^2\bar{x}_t^1\rangle = \mu_t^{21}\Sigma_t^{11}$, $\langle F_t^2\bar{x}_t^2\rangle = \mu_t^{21}\Sigma_t^{12}$, and $G_t^{11} = (\Sigma_t^{22})/(\det\Sigma_t)$.

In the model of the *E. c*oli bacterial chemotaxis, we have $N_t = 2T_t^m$ and

$$dP_t = \frac{1}{(\tau^m)^2}\frac{[\langle a_t^2\rangle - \langle a_t\rangle^2][\langle m_t^2\rangle - \langle m_t\rangle^2] - [\langle a_t m_t\rangle^2 - \langle a_t\rangle\langle m_t\rangle]^2}{\langle m_t^2\rangle - \langle m_t\rangle^2}dt$$

$$= \frac{1-(\rho_t^{am})^2}{(\tau^m)^2}V_t^a dt, \tag{39}$$

where $V_t^x := \langle x_t^2\rangle - \langle x_t\rangle^2$ indicates the variance of $x_t = a_t$ or $x_t = m_t$, and $\rho_t^{am} := [\langle a_t m_t\rangle^2 - \langle a_t\rangle\langle m_t\rangle]/(V_t^a V_t^m)^{1/2}$ is the correlation coefficient of a_t and m_t. The correlation coefficient ρ_t^{am} satisfies $-1 \le \rho_t^{am} \le 1$, because of the Cauchy-Schwartz inequality. We note that, if the joint probability $p[a_t, m_t]$ is Gaussian, the factor $1 - (\rho_t^{am})^2$ can be rewritten by the mutual information I_t^{am} as

$$1 - (\rho_t^{am})^2 = \exp[-2I_t^{am}], \tag{40}$$

where I_t^{am} is defined as $I_t^{am} \coloneqq \langle \ln p[a_t, m_t] \rangle - \langle \ln p[a_t] \rangle - \langle \ln p[m_t] \rangle$. This fact implies that, if the target system a_t and the other system m_t are strongly correlated (i.e., $I_t^{am} \to \infty$), no information flow exists (i.e., $dI_t^{\mathrm{tr}} \to 0$).

From the analytical expression of the transfer entropy (38), we can analytically compare the conventional thermodynamic bound [i.e., $\Xi_t^{\mathrm{SL}} \coloneqq -J_t^m dt/T_t^m + dS_t^{am} \geq J_t^a dt/T_t^a$] with the information-thermodynamic bound (12) for the model of *E. coli* chemotaxis [Eqs. (1) and (2) with $\bar{a}_t(m_t, l_t) = \alpha m_t - \beta l_t$] in a stationary state, where both of the Shannon entropy and the conditional Shannon changes vanish, i.e., $dS_t^{a|m} = 0$ and $dS_t^{am} = 0$. Thus, the conventional thermodynamic bound is given by the heat emission from m such that $\Xi_t^{\mathrm{SL}} \coloneqq -J_t^m dt/T_t^m$ and the information-thermodynamic bound is given by the information flow such that $\Xi_t^{\mathrm{info}} \coloneqq dI_t^{\mathrm{tr}}$. The information-thermodynamic bound is given by $dI_t^{\mathrm{tr}} = (1 - (\rho_t^{am})^2)[\langle a_t^2 \rangle - \langle a_t \rangle^2] dt/[4(\tau^m)^2 T_t^m]$. The conventional thermodynamic bound is given by $\Xi_t^{\mathrm{SL}} \coloneqq \langle a_t^2 \rangle dt/[(\tau^m)^2 T_t^m]$. From $-1 \leq \rho_t^{am} \leq 1$ and $\langle a_t \rangle^2 \geq 0$, we have inequality $\Xi_t^{\mathrm{SL}} \geq \Xi_t^{\mathrm{info}}$. This implies that the information-thermodynamic bound Ξ_t^{info} is tighter than the conventional bound Ξ_t^{SL} for the model of *E. coli* bacterial chemotaxis:

$$\Xi_t^{\mathrm{SL}} \geq \Xi_t^{\mathrm{info}} \geq \frac{J_t^a}{T_t^a} dt. \tag{41}$$

Supplementary References

[1]Sekimoto, K. *Stochastic Energetics* (Springer, New York, 2010).

[2]Seifert, U. Stochastic thermodynamics, fluctuation theorems and molecular machines. Rep. Prog. Phys. **75**, 126001 (2012).

[3]Cover, T. M. & Thomas, J. A. *Element of Information Theory* (John Wiley and Sons, New York, 1991).

[4]Ito, S. & Sagawa, T. Information thermodynamics on causal networks. Phys. Rev. Lett. **111**, 180603 (2013).

[5]Allahverdyan, A. E., Janzing, D., & Mahler, G. Thermodynamic efficiency of information and heat flow. J. Stat. Mech. (2009). P09011.

[6]Hartich D., Barato A. C., & Seifert U. Stochastic thermodynamics of bipartite systems: transfer entropy inequalities and a Maxwell's demon interpretation. J. Stat. Mech. (2014). P02016.

[7]Horowitz J. M. & Esposito M. Thermodynamics with continuous information flow. Phys. Rev. X, **4**, 031015 (2014).

[8]Shiraishi N. & Sagawa T. Fluctuation theorem for partially masked nonequilibrium dynamics. Phys. Rev. E, **91**, 012130 (2015).
